# Small, Smart, Fast, and Cheap: Microchip-Based Sensors to Estimate Air Pollution Exposures in Rural Households

**DOI:** 10.3390/s17081879

**Published:** 2017-08-16

**Authors:** Ajay Pillarisetti, Tracy Allen, Ilse Ruiz-Mercado, Rufus Edwards, Zohir Chowdhury, Charity Garland, L. Drew Hill, Michael Johnson, Charles D. Litton, Nicholas L. Lam, David Pennise, Kirk R. Smith

**Affiliations:** 1 Environmental Health Sciences, School of Public Health, University of California, Berkeley, CA 94720, USA; l.drew.hill@gmail.com; 2 Electronically Monitored Ecosystems (EME), LLC, Berkeley, CA 94710, USA; tracy@emesystems.com; 3 CONACYT—Instituto de Investigaciones en Ecosistemas y Sustentabilidad, Universidad Nacional Autónoma de Mexico, Morelia 58190, Michoacán, Mexico; ilse.ruiz@cieco.unam.mx; 4 Department of Epidemiology, School of Medicine, University of California, Irvine, CA 92697, USA; edwardsr@uci.edu; 5 Graduate School of Public Health, San Diego State University, San Diego, CA 92182, USA; zohir.chowdhury@sdsu.edu; 6 Berkeley Air Monitoring Group, Berkeley, CA 94704, USA; cgarland@berkeleyair.com (C.G.); mjohnson@berkeleyair.com (M.J.); dpennise@berkeleyair.com (D.P.); 7 Airviz, Inc., CREATE LAB, Carnegie Mellon University, Pittsburgh, PA 15213, USA; clitton@andrew.cmu.edu; 8 Department of Civil and Environmental Engineering, University of Illinois at Urbana-Champaign, Urbana, IL 61801, USA; nickllam@gmail.com

**Keywords:** PM_2.5_ monitor, stove use monitor, time-activity monitor, air exchange rate monitor, field validation, intrahousehold variation, household air pollution

## Abstract

Over the last 20 years, the Kirk R. Smith research group at the University of California Berkeley—in collaboration with Electronically Monitored Ecosystems, Berkeley Air Monitoring Group, and other academic institutions—has developed a suite of relatively inexpensive, rugged, battery-operated, microchip-based devices to quantify parameters related to household air pollution. These devices include two generations of particle monitors; data-logging temperature sensors to assess time of use of household energy devices; a time-activity monitoring system using ultrasound; and a CO_2_-based tracer-decay system to assess ventilation rates. Development of each system involved numerous iterations of custom hardware, software, and data processing and visualization routines along with both lab and field validation. The devices have been used in hundreds of studies globally and have greatly enhanced our understanding of heterogeneous household air pollution (HAP) concentrations and exposures and factors influencing them.

## 1. Introduction: Small, Smart, Fast and Cheap

To facilitate research on and evaluation of household air pollution (HAP) from solid fuel use in village settings in developing countries, we worked over the last 20 years to develop battery-operated, microchip-based devices to directly quantify a suite of parameters that had previously been assessed using expensive and otherwise inappropriate commercial monitors, or using questionnaires and direct observation, which are imprecise and can be intrusive in this context. Application of these devices also required, in addition to development of the technology itself, creating the software and data processing routines for handling the sometimes large datasets that result. 

Only one class of the devices described here actually measures air pollution itself, but all were developed in order to facilitate air pollution studies and each has important other uses as well. HAP derived from cooking is highly variable both spatially and temporally in village settings, where air pollution concentrations can vary by orders of magnitude within a few meters, with large diurnal variations due to daily cooking patterns, and in many regions, large seasonal variations. HAP levels also tend to have a high degree of intra-household variability, even over a few days, due to differences in stove use, ventilation, and time-activity patterns. Each of these covariates are of interest, therefore, not only since changes to them can serve as interventions to lower pollution exposures, but also because they affect estimates of long-term exposures needed for associating pollution to ill-health.

Additional motivation for the development of these sensing systems arose from the need to better quantify and standardize methods for measurement of microenvironmental (or ‘area’) concentrations and personal exposure to particulate matter with an aerodynamic diameter of 2.5 μm or less (PM_2.5_). This quantification allowed comparison of exposures from HAP with other sources of air pollution and enabled more sophisticated understanding of health effects, i.e. the development of exposure-response relationships. 

In the large set of studies performed in Guatemala as part of the RESPIRE project [1], for example, 50% of the variation in carbon monoxide (CO) exposures was explained by stove type, but all of the many dozen other household parameters then available were only able to account for another 10% [2]. Thus, there was substantial potential for exposure misclassification with only a few measurements per household—in other words, intrahousehold variability was high, so one or two exposure measurements may misestimate ‘true’ exposure. This can be addressed by conducting multiple measurements in each house, which was done in this study, but analysis shows that as many as five to seven 48-h measurements per home were needed to lower the chance of misclassification sufficiently to reliably link exposure to ill-health [2]. A long-term (approximately one-year) set of kitchen measurements (using the UCB-PATs, described in Section 2) in Guatemala showed further that many individual measurements over 24 h would be needed to be able to estimate long-term means accurately [3]. This in a setting where seasonal changes are muted due to the fairly small change in seasonal temperatures.

Another approach, however, is to reduce the unmeasured variability by measuring important exposure determinants including stove use (operational time of polluting sources), time-activity patterns (proximity to and frequency of contact with sources), and ventilation rates (which mediate the relationship between pollutant concentrations and personal exposures). Thus, during the Guatemala trial, we started in earnest the development of devices to objectively measure these three factors known to highly influence indoor air pollution concentrations and personal exposures. This allows the development of models in which there are fewer unmeasured factors, significantly enhancing long-term pollution exposure estimates.

Over the decades, four such exposure-related devices were developed and validated sufficiently by our group to become used by others, two now in widespread applications in more than 20 countries.

## 2. University of California, Berkeley−Particle and Temperature Sensor (UCB−PATS)

UCB-PATS uses the sensing chamber from a commercial home smoke detector that depends on natural air flow and diffusion to bring particles into its sensing chamber. It is a photometer that responds to an average concentration of particles. An infrared LED flashes at 2 s intervals; a photodiode picks up the response at 30-degrees forward scattering. The resulting amplitudes are averaged over a user-set interval (in minutes).

The original idea of adapting smoke alarm sensors as particulate matter (PM) monitors was developed in 1994 in the context of planned work in Guatemala as part of the search organized by the World Health Organization to find the best site for a hoped for randomized controlled trial on household air pollution. At the time, no relatively inexpensive commercial monitor existed that could be used for long periods of time in rural environments. Consultation with smoke alarm experts at the National Institute of Standards and Technology indicated adaptation of a commercial smoke detector was feasible, but no funding was found until 2000 with a grant from the then newly-formed Shell Foundation, an independent, non-profit charity addressing global development issues in the energy sector. 

The theoretical ability of the device to monitor PM_2.5_, the most important aerosol parameter for air pollution health studies, was evaluated in initial work that found simple aerosol theory could adequately explain the performance of the sensor [4]. The signal-to-noise ratio for particles between 150 and 500 nanometers in volume mean diameter was between 25 and 500 for mass concentrations between 0.50 and 16 mg/m^3^. Subsequent measurements were done in the laboratory and reported responses from prototype PATS units to gravimetric measurements, a TEOM (Thermo Fisher Scientific, Inc, Waltham, MA, USA), and a DustTrak 8520 (TSI, Shoreview, MN, USA) [5].These prototype units showed linear responses to across a range of concentrations of laboratory generated oleic acid aerosols (average R^2^ = 0.995 ± 0.005). Relationships with temperature and relative humidity were also characterized. 

The first field validation measurements were done in rural Mexico and Guatemala. Agreement across 19 monitors in co-location tests using wood burning aerosols in the field was highly linear (R^2^ = 0.99) and compared linearly with a collocated TSI DustTrak (Pearson R^2^ = 0.986) and with simultaneously collected gravimetric PM_2.5_ concentrations (R^2^ = 0.99). The monitors also estimated PM_2.5_ mass concentration well in wood burning kitchens (Pearson R^2^ = 0.89) and when tested in duplicate (Pearson R^2^ = 0.94) [6].

The original UCB-PATS monitor (at various times referred to as the UCB or UCB-PM) retained both the ionization and light-scattering chambers of modern smoke alarms (adapting an off-the-shelf model made by First Alert, Aurora, IL, USA), which offered substantial technical advantages for field particulate matter monitoring. The ionization detector is most sensitive to particle number and particles with diameters less than about 300 nm, where the infrared photoelectric scattering intensity decreases. Above 300 nm, the optical sensor’s angular intensity correlates reasonably well with mass concentration. Having two such distinct measurements was considered an advantage because it allowed for dynamic calibration of mass response based on size distribution over a broad spectrum of particle diameters and, in some cases, can assist in the identification of the source of pollution peaks when the size distributions are sufficiently different.

In these combination devices, it was hypothesized that the ratio of the increase in ion chamber signal to the increase in optical scattering signal could be used to define the appropriate sensitivity of the device for subsequent concentration determinations and adjustments [7]. The particle diameter at which the sensitivities were equal was about 220 nm; for particles with diameters less than this value, the ionization chamber yielded better concentration estimates, while for particles larger than this diameter, the infrared scattering chamber yielded better concentration estimates. In practice, this ratio was used to scale gravimetrically-derived mass coefficients to obtain more accurate correction factors across a range of particle diameters. Because number, surface, and mass sensitivities are functions of particle diameter, this ratio also provided a unique capability—by specifying the appropriate sensitivity to use, be it ionization or optical—to produce a more realistic and accurate concentration over a broad range of particle sizes.

Just as we were considering adding a control chamber for the ionization element to better control for the environmental sensitivities of the sensor, however, it became clear that—in spite of being exempted by the Nuclear Regulatory Commission as a smoke alarm containing less than 1 microcurie of americium-241 dioxide (^241^AmO_2_)—using the ion chamber for a new purpose would no longer be approved by campus and other health and safety offices (all activities were approved by UC Berkeley’s Committee for the Protection of Human Subjects). This was at least partly due to an incident in which a high school student, commonly known as the “Radioactive Boy Scout” [8], retrieved hundreds of smoke alarms to extract the isotope in an attempt to make plutonium. Although the risks posed by ^241^AmO_2_ are very low, this incident and increased attention to terrorism triggered by 9/11 resulted in authorities around the country treating smoke alarms with more caution. 

Subsequent devices that utilized only angular scattering at a single forward angle with no ionization chamber lost this unique capability, as well as most of the information relevant to particle size. Fortunately, these optical devices correlate well with PM_2.5_ mass, although they require calibration of mass response, similar to other light-scattering particle monitors. 

Standard home smoke detectors operate on a battery for long periods of time, which is a great advantage in rural developing rural settings, where electricity supplies are often intermittent or non-existent. As they are passive samples, field crews had to be adept at choosing locations where the monitor would be out of the way, would intercept natural air currents and exposure-relevant PM_2.5_ concentrations, and would meet standard placement protocols (1 m from the combustion source, 1.5 m above the floor). The data logger built around the smoke detector had a standard serial terminal interface to allow for setting the time and other sampling parameters and to offload and visualize stored real-time data. For broader acceptance, a great effort was made to build PC software that automated many of the procedures for setting up the device, and allowed automatic computation of averages and other statistical parameters for different time windows of interest. It made systematic archives of the data and enabled plotting and summarizing data in forms that are useful for larger project goals. 

With the formation of Berkeley Air Monitoring Group in 2008 to assist different groups globally in measuring and analysis of the impact of improved combustion stoves in India and Mexico [9,10,11,12,13], UCB-Particle and Temperature Sensor (PATS) became available commercially in a version relying solely on its optical sensor. UCB-PATS enjoyed a period of popularity as it provided many groups a means to obtain data on air pollution at reasonable cost (~US $500) and without requiring extensive access to expensive laboratory facilities and supplies—including costly and delicate filters, μg-precision balances, and sub-zero freezers—needed to support gold-standard monitoring. It has been used in studies in at least a dozen countries (a partial listing of studies using the UCB-PATS, organized by country in which the studies were done, can be found at http://www.kirkrsmith.org/s/UCB-History-May-2015-ycsl.pdf). 

The UCB-PATS was also integrated with a real-time audio-visual feedback system for wildland firefighters [14]. The system provided firefighters, who typically do not wear breathing masks or respirators due to long and physically demanding work periods, with a sound and light alarm when the particulate levels reached given thresholds, therefore providing an indication of when breathing protection should be judiciously worn. The system suggested that up to three-fourths of particulate exposure could be avoided using a 1 mg/m^3^ alarm threshold, conveyed to the firefighter via quickly discernable, level-dependent visual feedback.

Although remarkably robust (some are still in service), the limitations of the UCB-PATS became more troublesome over time, particularly the need to post-process data to address frequent shifts in the baseline response (due to either temperature dependence or degradation of the optics over time) and its use of growingly antiquated connection hardware (9-pin DB9 Serial Port) and computer software. In 2013, First Alert ceased production of the smoke alarm model that had been the basis of the UCB-PATS and in 2014, manufacture of the UCB-PATS ended at the time when a more advanced monitor, the PATS+, was being developed.

As the UCB-PATS reached its point of obsolescence, we initially thought to continue with a different commercial smoke detector. However, the sensing elements of new smoke detector models were more highly integrated and thus more difficult to disassemble for cleaning and to modify for our purposes as compared to past models. New options become available at the time, led by the Dylos 1100 monitor (Dylos Corporation, Riverside, CA, USA, described below in connection with the BAIRS project). The Dylos, however, was larger and more power hungry than the UCB-PATS and saturated at levels well below what is commonly seen in village households. Despite these limitations, the Dylos has proven useful for other applications in the developed world (discussed briefly in Section 4). 

At that point in history (2013), several other off the shelf commercial smoke or dust detector modules became readily available on the market. In particular, there are the PPD42NS and GP2Y1014 modules manufactured by Shinyei (Kyomachi, Chuo-ku, Kobe, Japan) and Sharp (Osaka, Osaka Prefecture, Japan), respectively, directed primarily at the air conditioner and air filter markets and sold through many distribution channels at a cost commensurate with a home smoke detector. 

## 3. PATS+

Funded by a U.S. Department of Energy grant in 2012, Berkeley Air Monitoring Group, along with members of our research group, worked to build a next-generation PATS, the PATS+. PATS+ is smaller than UCB-PATS, performs more on-board processing via a multicore Propeller microcontroller (Parallax, Inc., (Rocklin, CA, USA) that uses a modified version of the commercially available Sharp GP2Y1014 sensor. PATS+ is now being sold by Berkeley Air and is used by groups around the world (as of June 2017, the selling price was approximately 500 USD per monitor). 

For PATS+, we settled on the Sharp GP2Y1014 optical sensor due to its small physical form factor, its linear response to particulate matter, and its relatively low power requirements. It is similar to a standard photometer, looking at IR light scattered at a 30° forward angle within a relatively open particle path between lenses facing the LED and the photodiode. One of the main goals for deployments where biomass cooking occurs is a wide dynamic sensing range, as mass concentrations of PM_2.5_ can vary between lower limits of 10 μg/m^3^ and up to more than 30,000 μg/m^3^. The PATS+ incorporates precision control over the current and timing that drives the LED in the Sharp GP2Y1014 in order to extend the measurement range beyond its standard specification. The upper limit of detection claimed by the manufacturer (~1000 μg/m^3^), and as reported in numerous evaluations using the standard voltage drive circuit, is largely due to overdrive of the Sharp electronics, and not due to obscuration of the optical path or saturation of the photodiode. The PATS+ firmware drives the Sharp LED at two distinct intensities, enabling high-sensitivity and low-sensitivity measurement channels. Output from these two channels is integrated in the final datastream. Within PATS+, a small fan is used to maintain constant air flow, enough to assure air exchange within the detection chamber and increased sensitivity while still allowing a 2-day deployment on an internal rechargeable lithium polymer battery. Battery life can be extended with an external battery pack. There is also provision for a COA4 electrochemical carbon monoxide sensor Alphasense, (Great Notley, Essex, United Kingdom) as well as measurements of air temperature, humidity, and motion. 

**Figure 1:**
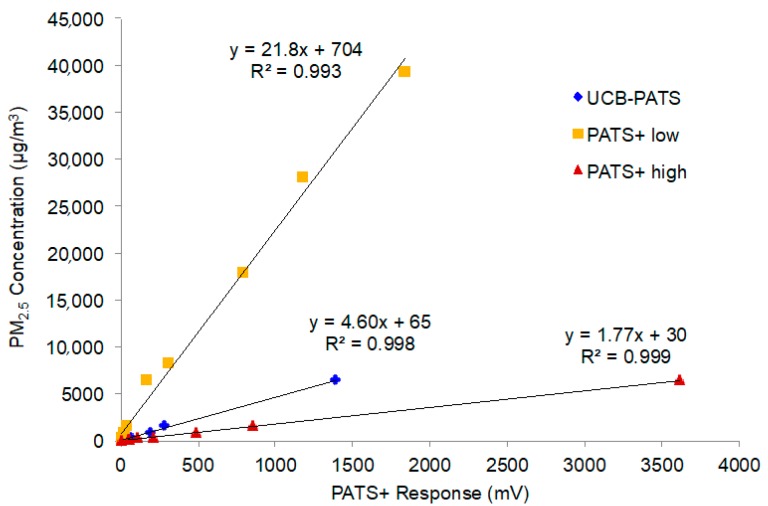
Relationships between PATS+ photoelectric response (low and high sensitivity channels in mV) and PM_2.5_ concentrations based on laboratory chamber tests. Each point represents 15-min mean concentrations during a particle exposure event. The Y-axis is based on calibrated DustTrak readings.

A large part of the development effort for the PATS+ at Berkeley Air went into the firmware and software to support the so-called Platform for Integrated Cookstove Assessment (PICA). PICA improves and extends earlier monitor software, simplifies the configuration and operation of the PATS+ at field sites, and allows field staff to offload data from many different measurement devices and easily merge and visualize them together. Figure 1 shows the linear relationships of the low and high sensitivity channels of the PATS+ with a gravimetrically-adjusted TSI DustTrak II Aerosol Monitor 8530 during laboratory chamber tests. The mean results for three UCB-PATS instruments (the predecessor of the PATS+) are also shown for comparative purposes. The strong linear correlations (R^2^ > 0.99) indicate that the PATS+ photoelectric signals responded consistently and predictably to increasing concentrations of particulate matter, as well as or better than the UCB-PATS. The two sensitivity channels allow the PATS+ to measure a very wide range of particle concentrations, with the low sensitivity channel handling high PM concentrations and the high sensitivity channel providing increased resolution at low PM concentrations.

**Figure 2:**
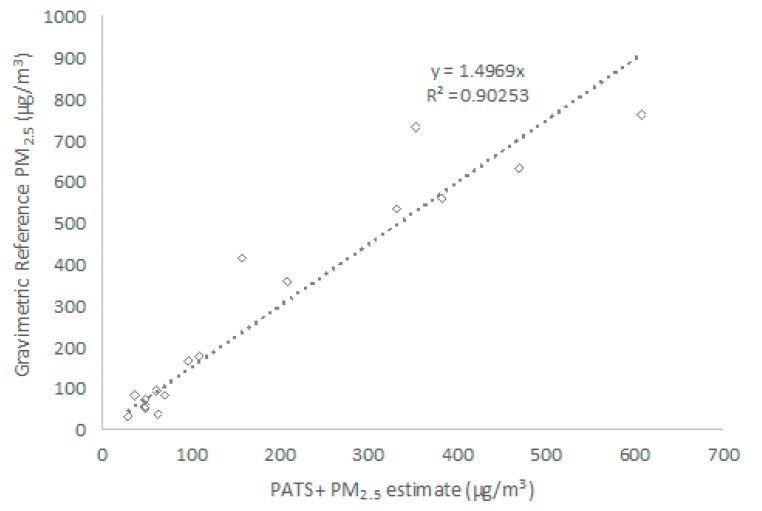
Correlation between the PATS+ estimate of PM_2.5_ (μg/m^3^) and collocated gravimetric samples of PM_2.5_ (μg/m^3^) from a PATS+ field performance test in the kitchens of families using traditional wood burning cookstoves in Alotenango, Guatemala. PATS+ units were individually calibrated against wood smoke in the lab.

Figure 2 shows the correlation between the PATS+ and gravimetric samples from 48-h collocations in kitchens of families using traditional wood burning cookstoves in Alotenango, Guatemala. The strong linear correlation (R^2^ > 0.90) indicates that the PATS+ photoelectric signals responded well across the range of particulate matter concentrations under real-world conditions. PATS+ output did consistently underestimate actual concentrations, but this can be addressed with in-field calibration. 

**Figure 3:**
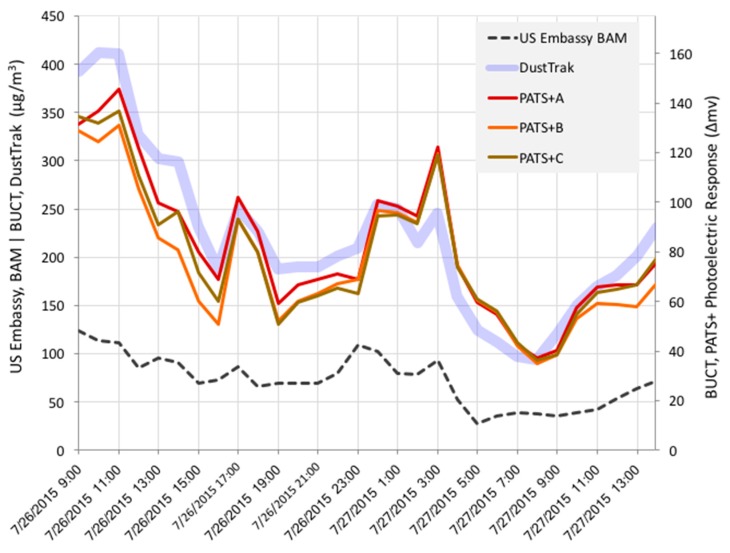
Hourly average trace of responses from (1) co-located PATS+ units (change in mV from the photoelectric chamber), (2) a DustTrak (μg/m^3^, uncorrected PM_2.5_) near Beijing University of Chemical Technology (BUCT), and (3) the US Embassy BAM (μg/m^3^, PM_2.5_) located approximately four kilometers away.

**Figure 4:**
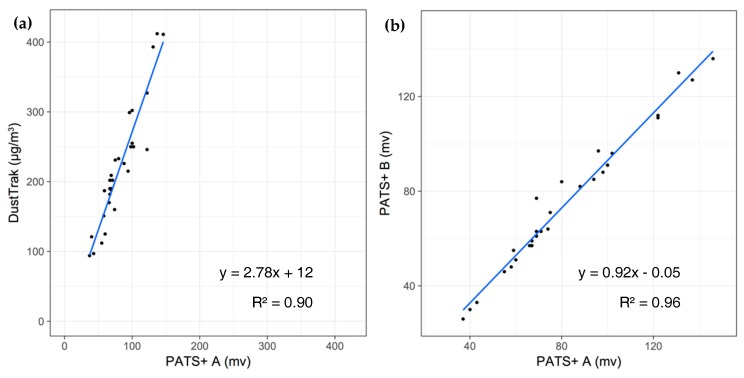
Correlation of (**a**) PATS+ and DustTrak and (**b**) PATS+ intra-instrument comparison during the Beijing monitoring period (see Figure 3). Each data point represents a one-hour average.

The PATS+ has also been evaluated for applications in polluted urban environments. Figure 3 depicts an hour-by-hour trace of three PATS+ co-located with a DustTrak near the Beijing University of Chemical Technology (BUCT). For reference, the PM_2.5_ concentrations reported by the US Embassy’s BAM (Met One, Grants Pass, OR, USA), located approximately 4 km away [15,16], are also shown. The DustTrak and PATS+ units clearly track well with each other and the PATS+ monitors track well with one another even though the two devices were not calibrated in this study (only raw output data shown), as supported by the strong correlations shown in Figure 4. Assuming that the absolute BAM concentrations (~35–125 μg/m^3^) are indicative of what the DustTrak and PATS+ were measuring, the PATS+ performance appears to be suitable for urban environments where PM_2.5_ pollution is a major concern.

## 4. Particle Monitors with Other Sensors

We have also developed and tested Berkeley Aerosol Information Recording System (BAIRS), a benchtop monitor that relies on a more sophisticated, but still consumer-based optical particle counter, the Dylos Air Quality Monitor (DC1100 and DC1700). A controlled air flow brings particles through a laser beam where pulses of scattered light are transduced into electrical pulses, the amplitude of which are classified into bins correlated to different particle sizes. 

The limit of detection of BAIRS was less than 1 μg/m^3^ with 1 μg/m^3^ resolution. This high sensitivity is due in part to a beam that passes above a large photodetector; scattering events are thus captured across a range of angles. BAIRS bins particles into several size ranges (<0.5 μm, 0.5–1.0 μm, 1.0–1.7 μm, 1.7–2.5 μm, and >2.5 μm) and when size distributions are sufficiently different can estimate the mass concentration of particles from different sources. Its response rounds off and saturates at mass concentrations greater than approximately 10.0 mg/m^3^. For the BAIRS, Dylos Corporation programmed a customized firmware version (2.05 w) that added size bins and additional commands that allow control by an external microcontroller of the data acquisition timing and the power supply. In an ambient roof-top test of the BAIRS and a commercial light scattering particle monitor, the BAIRS response tracked well with the commercial monitor; daily means were within 80% of each other [17].

A similarly modified version of the Dylos was created by Klepeis and others and was utilized for projects related to detection of tobacco smoke. This version, in addition to a datalogger, contained provisions for wireless communication with a laptop and software to visualize collected data. Participants in these studies received audiovisual feedback related to detected smoke levels [18].

A former doctoral student in our group successfully developed and tested a related device, the Portable and Affordable Nephelometric Data Acquisition (PANDA) system, which is based on a different sensor, the Shinyei PPD42NS. The Shinyei sensor leaves its LED light source on continuously and tracks fluctuations in scattered light, not as individual particles, but in aggregate over a period of time. Multiple PANDAs were calibrated using 1-h and 24-h PM_2.5_ data from a class III US EPA Federal Equivalent Method (FEM) PM_2.5_ β-attenuation monitor at a regulatory monitoring site in Oakland (CA, USA). Linear corrections explained 60% of the variance in 1-h reference PM_2.5_ data and 72% of the variance in 24-h data. Performance at 1-h integration times was comparable to commercially available optical instruments costing considerably more [19].

## 5. Stove-Use Monitoring System (SUMS)

Based on insights from a visit to a project in India using monitors to keep track of usage of Compact Fluorescent Light (CFL) bulbs for a carbon credits project in 2007, we innovated approaches to directly measure the use of stoves by deploying data-logging temperature sensors, which enable much longer, unobtrusive, and accurate monitoring of cooking behavior and “stacking”−combined use of more than one stove-fuel in a kitchen [20]. 

**Figure 5:**
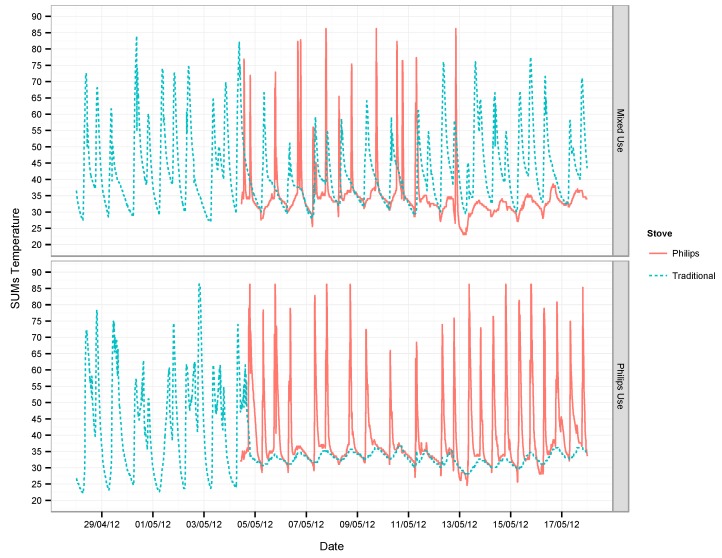
SUMs data from households with different usage patterns. Panels show temperature traces (degrees C) for a traditional stove (blue dashed line) and for the Philips, a fan-assisted intervention stove (solid red line). The top panel shows a household where the stove was incompletely used, with consistent use of both the traditional and intervention stoves. The bottom panel shows a household that moves from their traditional stove to the advanced cookstove. Adapted from [21].

Motivations to directly measure stove usage included: (1) the need to reduce exposure misclassification based on observed or reported stove type and (2) the promise of objectively verifying stove usage for carbon projects. At the same time, by 2007, there was enough evidence from Guatemalan and Mexican studies that accurate exposure assessment of HAP required a better understanding of the stove adoption process. Introducing a new cooking technology into homes often led to its abandonment after implementation and/or to the use of more than one stove-fuel combination, all of which critically shaped the actual impacts of the interventions. Figure 5 shows the types of data generated by SUMs.

Stove Use Monitors (SUMs) initiated a new era of research to understand the long-term dynamics of adoption, to link laboratory tests to field performance through usage patterns and, for the first time, to provide actionable data to understand and respond to the household needs and preferences that make people use or not use intervention stoves and traditional open fires. 

Fabrication of customized devices to measure temperature (or electrical current for stoves with a fan) that were small, rugged, radio-enabled for remote data offload, power-efficient enough to operate for months, and embeddable into the stove was not feasible at the time at target prices lower than the cost of the stoves, even at volumes of hundreds of thousands. Instead, we used the off-the-shelf coin-sized iButton temperature dataloggers (model numbers: DS-1922T, DS-1921G, DS-1921L Maxim IC, San Jose, CA, USA) as the first SUMs and relied on Palm PDAs, and later on laptop computers, for launching sensors and visualizing and downloading data in the field using a probe.

Ilse Ruiz-Mercado, now at the National Autonomous University of Mexico, undertook field implementation of the SUMs in the CRECER study, establishing a methodology for placement, data management, and signal analysis algorithms. The 3-year set of measurements in the Guatemala trial demonstrated that accurate, long-term, unobtrusive quantification of stove and open fire use was possible by obtaining metrics for cooking events in terms of days and hours of usage. It also showed that with robust sample designs, adequate sensor placement protocols, and careful data collection and management procedures, the SUMs could be a cost-effective monitoring tool. Longitudinal analysis of daily statistics with mixed models resolved seasonal fluctuations in usage as small as 3%. In this particular epidemiological trial recalled questionnaires of stove use frequency and duration were consistent with SUMs data, and partition of variances confirmed the stable day-to-day cooking behavior for this population where between-household differences in measured usage contributed 76% to the total variability.

iButtons can store between 1–3 months of data logging at sampling frequencies of 5–20 min. With proper placement, the non-replaceable battery lifetime was around 1.5 years. Data loss was 10% in the Guatemala study, mainly due to overheating or water dripping into the sensors. Both of these were later overcome using mounts and holders, but a standard solution to ensure sensor integrity while still capturing strong signals remained a challenge during those years, particularly for open fires that changed location around the home. This formative initial research was described in a series of papers by Ruiz-Mercado et al. [22,23,24,25].

Berkeley Air Monitoring Group now sells sets of iButton-based SUMs and associated software for others to use. As of June 2017, the selling price for a package of 100 1921G iButtons (−40 to 85 C measurement range) was approximately $2800 with associated software and cables. It is fair to say, we believe, that since our first use, SUMs in some form are now seen as being essentially required in nearly all intervention and health-related studies involving field use of stoves. iButtons have been used in studies in India, Nepal, China, Ghana, Guatemala, Mexico, Kenya, Sudan, Cambodia, Lao, and Mongolia by researchers, NGOs, and governmental agencies ([21,26,27,28,29,30]; see the current list at Berkeley Air Monitoring Group’s website: http://berkeleyair.com). 

iButtons and other similar devices fulfilled the needs of several studies. However, use of iButtons imposes some limitations making their utility by non-research groups challenging, including the need to enter monitored homes and the difficulty of processing raw data into meaningful and standardized metrics of stove use. As a result, we have evaluated a number of other commercially available temperature loggers for use as SUMs, including infrared (IR) thermometers and low-cost datalogging thermocouples. EME Systems has also created a multi-probe K-type thermocouple datalogger (the “kSUM”’), enabling monitoring of multiple combustion sources with a single logger and easing placement concerns due to much higher maximum temperature limits. This version has been used successfully to track biomass and LPG stoves in Peru [31]. We have used the SUMS in assessments of stoves both in short and longterm applications and also now use custom-built SUMs for specific project needs. We additionally created a ‘wireless’ SUMs that processes data on board and transmits summary usage metrics (discussed below). 

## 6. Wireless SUMS

With funding through an innovation prize from Vodafone Americas Foundation in 2010, we developed a wireless version of the SUMS (wSUMS), which allows data on stove use to be downloaded unobtrusively without entering the household. The goal is to verify stove adoption and potential use as part of larger-scale carbon projects. The wSUMS consists of a single, rectangular transmitter box from which leads are drawn and placed on different hearths and stoves throughout the home. A single transmitter can be connected to three combustion sources. The unit detects a differential temperature (automatically corrected for ambient temperature). At regular intervals, a fieldworker can walk through a neighborhood or block with a receiver, which activates wSUMS transmitters and initiates data transfer. Unlike the iButton-based SUMs, wSUMS sends compressed data consisting of a single line of summary parameters—including daily time above a threshold temperature, daily degree-days above threshold, and overall statistics for the duration of each deployment. Data transmission is rapid and efficient and relies on short-range IEEE 802.15.4 radio. Transmitters must be well-placed, with consideration of radio propagation, for the receiver to successfully obtain data packages without entering homes. Analysis challenges are reduced by the onboard processing of logged temperature into usable metrics. Although field tested in Mexico and India, it did not find as wide an application as we had hoped largely due the sudden collapse of the global carbon price at about the time of the end of the Kyoto Treaty. Thus, the main market we had envisaged has not yet come to pass [32].

Nevertheless, in collaboration with Daniel Wilson of Geocene (Vallejo, CA, USA), we are now doing the fieldwork at two sites in India needed to deploy a different type of wireless SUMs as part of the large 4-country randomized controlled trial being funded by the United States National Institutes of Health and the Gates Foundation (clinicaltrials.gov #NCT02944682). The system is composed of a data-logging thermocouple that is downloaded and programmed using a mobile phone or laptop. Fieldworkers can download data from outside the home (if needed), though downloading in home allows fieldworkers to interact with households to further inquire about usage patterns. 

## 7. Time-Activity Monitors (TAMs)

We have developed a simple, inexpensive, ultrasound-based system for monitoring the amount of time spent by different household members in specific household locations. The monitor uses ultrasound to measure the amount of time individuals spend in various household micro-environments (HME, e.g., kitchen, bedroom, living room) and in the vicinity of important sources of air pollution. Individuals wear a matchbox-sized ultrasound pulse-emitting tag which is detected by receivers placed within distinct HMEs.

Ultrasound has the advantage that it is well confined by walls, which makes it possible to localize the tag and its bearer with accuracy within those walls, and thus to assess their individual exposure to pollutants in the room. The alternatives have disadvantages. Radio (such as car door openers) does penetrate walls, so a signal detected does not necessarily imply exposure, and light (such as TV remotes), while confined, is not omnidirectional enough nor does it penetrate simple clothing well enough to be practical. 

In TAMS, the 40 kHz ultrasound is radiated by small (25 g) locator transmitters carried by participants, and a locator receiver mounted on a wall in the room of interest decodes and logs the IDs received from the individual transmitter tags. The IDs are encoded as a set of 15 possible binary patterns, and those are sent at random intervals in order to avoid repeated collisions. An algorithm in the receiver is configured with thresholds that separate noise and collisions from true detections. The individual bit lengths had to be at least 0.1 s long in order to allow for reverberations to die out in the typical room before the onset of the subsequent bit. 

**Figure 6:**
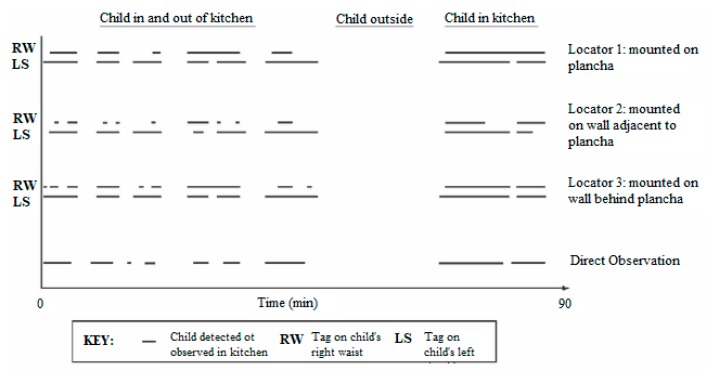
Direct observation vs. UCB-TAMS data for one mealtime observation. The lowest set of dashes represent time that the child was in the kitchen based on fieldworker observation—the ‘gold standard’. Adapted from Piccolo-Allen [33].

We described a series of laboratory test of detection success based on the number of tags present, the type of cloth holder for the tag, direct versus reflected signals, the effects of extraneous ultrasonic noise, and the possibilities of false detections near doors and windows [33]. The detection was reliable for up to 4 or 5 tags present in a room, which was acceptable when the main participants were a mother and a couple of children. The field work in Guatemala showed a 90–95% accuracy of predicting time-weighted average location in the kitchen and minute-by-minute accuracy of 80–85% (Figure 6). The sensitivity was 86–89% and specificity was 71–74% for one-minute readings on children compared to the gold standard of direct observation by trained fieldworkers.

In late 2013, our group received several small grants to work with EME Systems (Berkeley, CA, USA) to modernize the TAMS as a proof of concept and explore alternate use cases relevant to development economists. Development focused on: (1) enhancing the specificity of the tag-receiver combinations, to decrease the false positives noted during preliminary testing, (2) use of a new real-time clock to quash outstanding bugs with date-time logging, (3) enhanced firmware to allow customization of logging parameters, and (4) writing logs to SD cards, as opposed to on-device memory. 

## 8. Air Exchange Rate Monitors (ARMs)

Our group pioneered the use of carbon monoxide (CO) as a tracer gas to measure air exchange rates (AERs) in village households in the 1980s [34] as an indicator of ventilation. We have typically used a smoldering fire to generate a portable source of CO, then, after removing the source, measured how quickly it was eliminated from the room. This approach, however, was cumbersome, time-consuming, and potentially exposes the research team and householders unnecessarily to CO, a toxic gas.

**Figure 7:**
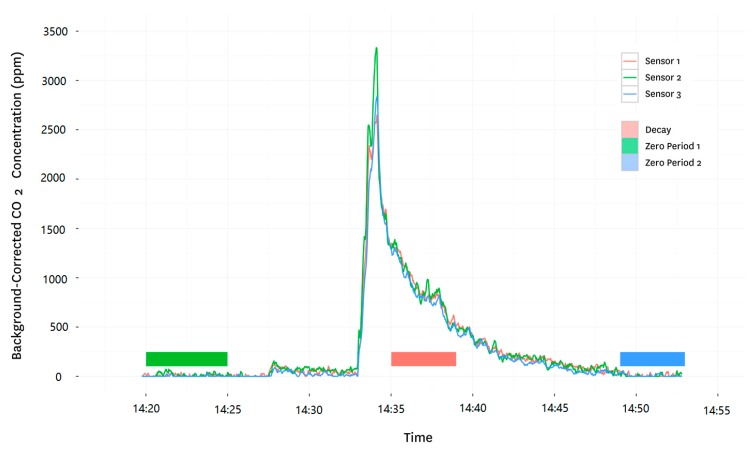
Sample ARMs trace from a single measurement period in a rural Nepali kitchen. A line was fit to the long-transformed values during the linear decay period (marked on the graph by the pink square) to estimate air changes per hour.

We have now developed a more formal system relying on carbon dioxide as a tracer that allows repetitive measurements under the same conditions in one or many households. It relies on a cylinder of carbon dioxide (CO_2_) of the type commonly available around the world because of its use to make beverages. The system consists of a data-logger with between one and four non-dispersive infrared (NDIR) CO_2_ sensors attached on individual wire arms of approximately 20 feet in length. Each sensor is suspended on a tripod and placed at a standardized position in the room. Inhabitants are asked to vacate the room temporarily and a bolus of CO_2_ is released into the room. A line is fit to the background corrected log CO_2_ concentration during the decay period; its slope is the AER in air changes per hour (ACH, Figure 7). Analysis of over 3000 AER measurements made in Nepal is ongoing [35].

## 9. Discussion

The aforementioned monitoring systems—including hardware and software—were developed over long periods, but have proven transformative in assessments of the impacts of household energy. Prior to their existence, the field relied heavily on survey and questionnaire-based measures of stove and fuel use. The ability to transition to objective sensing systems has dramatically altered our understanding of stove use, of kitchen and living area pollutant concentrations, and of personal exposures, and has helped elucidate emerging issues, like stove stacking.

Monitoring air pollution in remote, rural settings can be challenging. Our monitoring systems must optimize between conflicting goals of ruggedness, precision, and long battery life, to name a few, and must be small, quiet, unobtrusive, and relatively low cost. Years of work have led to a suite of sensors that enable deployment of many sensors in many homes—greatly enhancing our understanding of the wide variability within and between homes, regions, and countries. This has started to upend the previous paradigm of single, 24-h measurements in households. 

### 9.1. The Development Pathway

A number of lessons can be gleaned from our 20+ year experience developing such monitors. Here we note only four. First, the transition from initial concept, to prototype, to lab verification, and then to field validation can be long, with each step taking considerable additional thinking and work. In our case, we did not have the advantage of serious external funding for any of the devices except for the transition from the UCB-PATS to the PATs+, which thus required us to take advantage of student theses, existing projects, and ongoing curiosity to move forward.

Second, since all the monitors rely on datalogging to store the sometimes quite large data sets that are generated, developing the needed data cleaning and processing protocols was integral to producing usable devices in the end. This also took much time and trial and error with field data and are now moving to becoming web-based.

Third, as the field developed, there came to be more interest among other groups in acquiring the devices and, for some, in receiving technical support for data analysis. The university, however, is not well set up to sell and service devices on a routine basis but the development of the local independent company, Berkeley Air Monitoring Group (BAMG), allowed us to shift these activities to it and thus provide a way to disseminate devices to a range of groups around the world on a commercial basis. Finally, it has become clear that training is often required for many groups to use the devices well. Our group, BAMG, and other groups now routinely help with such training, which nevertheless adds costs.

### 9.2. Next Steps & Considerations

More than two decades ago, we recognized the need to transition from questionnaires and expensive, ill-suited air pollution monitors to a set of custom tools to better enhance our ability to estimate exposure. While we have made much progress on this front, we identify a few areas of continuing work, by our group and others.

First, we acknowledge a need to transition from individually managed sensors to cloud-enabled devices, leveraging reasonable cellular data penetration around the world, including in rural areas. Fieldworker burden could be substantially alleviated by enabling remote monitoring of some parameters; recent advances in short-range radio technology enables, for example, using a mobile phone to download sensor data outside of a home and transmit it instantly or when back in range of network access to a centralized server. This type of realtime analysis could enable novel feedback to households that are, for example, not using an intervention stove. 

Second, for air pollution sensors in particular, the issue of requiring periodic and thorough calibration remains. Currently, many PM sensors must be calibrated against a gravimetric sample, requiring careful filter handling and significant, if occasional, laboratory support. Calibration should occur at least once against the aerosol of interest. Calibration factors change with fuel and combustion characteristics; for instance, correction factors for the same fuel burned in an open fire and a chimney stove have been shown to vary substantially [36]. As fuel and stove use may change by season, this indicates a need to calibrate periodically. Laboratory calibration against a consistent fuel source enables between device comparison, ensuring responses are comparable, but is not a substitute for infield colocation with a gravimetric sampler.

A number of promising methods—leveraging advanced statistical techniques and/or more detailed characterization of particle size—may help reduce the frequency of this calibration. One technique of interest explored during the initial UCB-PATS work is dynamic calibration and particle sizing through analysis of concurrent ionization and optical sensor output. This work was limited in part by the legal barriers to handling and distributing Americium. However, it is conceivable that recent advances in dual-wavelength optical smoke-detection chamber technology may be used in a similar manner to discern particle size. For example, members of our group have explored the PM_2.5_ measurement abilities of the dual wavelength smoke-detection chamber of the Nest Protect ‘smart’ smoke detector [37] which utilizes an optical chamber with two LEDs of different wavelengths (IR and blue visible light) at forward scattering angles of 45 degrees. IR and blue signals correlated strongly (adjusted R^2^ > 0.99) with filter-adjusted readings from a DustTrak II at levels as high as approximately 15 mg/m^3^ with typical noise (likely due in part to the crude hardware used to access raw sensor signal during testing, which can be considerably improved) measured between 7–30 μg/m^3^. Although not validated experimentally, theory indicates that juxtaposition of simultaneous output from the two (or more) wavelengths may enable particle sizing over a limited range in a manner similar to that previously explored with the dual ionization-optical chamber of the original First Alert detector utilized in the UCB-PATS. This could also facilitate dynamic calibration. The Nest Protect can be purchased off-the-shelf for approximately $120 (USD), which is more expensive than the components used in the UCB-PATS or the more-current PATS+, but not prohibitively so given the promise of added calibration and sizing capabilities.

Moreover, researchers should evaluate the ability of smart smoke detectors already installed to monitor, process, communicate, and potentially calibrate PM_2.5_ concentration data [37]. While this requires cooperation of many stakeholders and more investigation, the potential for an in-situ indoor monitoring network of these devices could reduce exposure misclassification (a perennial problem in detecting results during epidemiological studies) at both the individual and population levels. Given the cost and availability of smart smoke detectors, a system like this is currently limited to wealthier urban regions. More work is needed to characterize the resolution of smart smoke detectors at low concentrations experienced during non-alarm periods. On the other hand, one could envision a future scenario where the health benefits from improved air quality monitoring of a converted smart smoke detector (buttressed by the added value of fire risk prevention) justifies rural dissemination campaigns in areas were internet connectivity is reliable.

Another potentially viable technique utilizes a single wavelength but measures intensities at different angles to elucidate particle size. Some limited data [38] indicated the potential of this technique to discriminate between respirable coal dusts with relatively large diameters and ultrafine particles emitted from a diesel engine. Theory indicates that this technique has application over a broader range of particle sizes than the dual wavelength approach and, in addition, is less sensitive to the optical properties of the particles, such as the index of refraction. It is possible that a device that utilizes both multiple wavelength light sources and multiple angle scattering could be the ultimate solution for inexpensive, personal particulate monitors. 

Third, given limited resources and time, more work is needed to understand how frequently pollutant sampling and stove usage monitoring needs to occur to adequately capture variability to predict exposure. Part of this question could be addressed by deploying the full suite of sensors described above in a sample of households for a period of time and developing models using modern statistical learning techniques to determine what parameters are most important, thus informing future sampling strategies.

Finally, advanced statistical techniques should be employed to leverage inexpensive sensor technologies for even more cost-effective reductions in exposure measurement misclassification. Hill explored these possibilities in his doctoral dissertation, wherein random forests, neural nets, and ensemble methods—among others—are used to reliably model population-wide exposures from easier-to-perform measurements of environmental variables and survey indicators [37]. From only a small number of measurements, actual exposures in a larger population were predicted using these methods with low cross-validated bias and a cross-validated coefficient of determination as high as R^2^ = 0.49. Such modeling methods could be combined with an arsenal of small, smart, fast, and cheap sensors to enhance the cost-effectiveness of large-scale population exposure estimation in both rural and urban settings.

Taken in total, these suggestions provide a compelling agenda for the modernization of air pollution exposure assessment within the context of rapid advancements in commodity sensors occurring as part of the larger consumer electronics zeitgeist [39]. Advancement along these paths will require innovation from and collaboration between engineers, epidemiologists, statisticians, and exposure scientists.

## 10. Conclusions 

Today, there is much more interest in HAP studies than even in the recent past with, for example, many large randomized controlled trials either underway or preparing for publication at sites around the world [40,41,42,43,44]. This has greatly increased the need for pollution monitoring equipment as most of these trials have recognized the value of good exposure assessment. Although still usually suffering from a relative lack of funding for the exposure assessment component, in a relative sense, there is less need for extremely inexpensive equipment than in the past for the largest of these studies. Thus, there are some 3–4 other small pollution monitors now available for purchase or to be borrowed on a collaborative basis from other groups in the U.S. and U.K. Each with its own pluses and minuses, although none as inexpensive as those we still make available.

There are dozens of less well-funded groups doing fieldwork around the world, however, that still need devices, like those we have developed, that can be deployed in village settings with the ability to measure extremely high as well as low pollution levels, that can be easily cleaned without damage, and that work without access to reliable power. Although they still require periodic calibration against gravimetric measurements, our devices do not require extensive laboratory backup. Indeed, low-cost is important for many groups, since it facilitates taking multiple measurements over time in each household. When combined with measurements of important cofactors, such as stove use, time-activity, and ventilation, these measurements can be used to better understand and describe long-term average concentrations and exposures.

## Abbreviations

The following abbreviations are used in this manuscript:

^241^AmO_2_	americium-241 dioxide
ACH	air changes per hour
BAIRS	Berkeley Aerosol Information Recording System
BAM	β-attenuation monitor
BAMG	Berkeley Air Monitoring Group
BUCT	Beijing University of Chemical Technology
CFL	compact fluorescent light
CO	carbon monoxide
CO_2_	carbon dioxide
CRECER	Chronic Respiratory Effects of Early Childhood Exposure to Respirable Particulate Matter
FEM	federal equivalent method
HAP	household air pollution
HME	household micro-environments
IR	infrared
kSUM	K-type thermocouple stove use monitor
LED	light-emitting diode
LPG	liquefied petroleum gas
NGOs	non-governmental organizations
PANDA	Portable and Affordable Nephelometric Data Acquisition
PICA	Platform for Integrated Cookstove Assessment
PC	personal computer
PM	particulate matter
PM_2.5_	particulate matter with an aerodynamic diameter of 2.5 μm or less
RESPIRE	Randomized Exposure Study of Pollution Indoors and Respiratory Effects
SUMS	Stove Use Monitoring System
TAMs	time-activity monitors
TEOM	tapered element oscillating microbalance
UCB-PATS	University of California, Berkeley—Particle and Temperature Sensor
wSUMS	Wireless Stove Use Monitoring System
